# Acute administration of beta-caryophyllene prevents endocannabinoid system activation during transient common carotid artery occlusion and reperfusion

**DOI:** 10.1186/s12944-018-0661-4

**Published:** 2018-02-05

**Authors:** Laura Poddighe, Gianfranca Carta, Maria Pina Serra, Tiziana Melis, Marianna Boi, Sara Lisai, Elisabetta Murru, Laura Muredda, Maria Collu, Sebastiano Banni, Marina Quartu

**Affiliations:** Department of Biomedical Sciences, University of Cagliari, Cittadella Universitaria di Monserrato, Monserrato, (CA) 09042 Italy

**Keywords:** Bilateral common carotid artery occlusion, Endocannabinoid system, PPAR-alpha, Beta-caryophyllene, Cerebral cortex, Plasma

## Abstract

**Background:**

The transient global cerebral hypoperfusion/reperfusion achieved by induction of Bilateral Common Carotid Artery Occlusion followed by Reperfusion (BCCAO/R) has been shown to stimulate early molecular changes that can be easily traced in brain tissue and plasma, and that are indicative of the tissue physiological response to the reperfusion-induced oxidative stress and inflammation. The aim of the present study is to probe the possibility to prevent the molecular changes induced by the BCCAO/R with dietary natural compounds known to possess anti-inflammatory activity, such as the phytocannabinoid beta-caryophyllene (BCP).

**Methods:**

Two groups of adult Wistar rats were used, sham-operated and submitted to BCCAO/R. In both groups, 6 h before surgery, half of the rats were gavage-fed with a single dose of BCP (40 mg/per rat in 300 μl of sunflower oil as vehicle), while the second half were pre-treated with the vehicle alone. HPLC, Western Blot and immunohistochemistry were used to analyze cerebral cortex and plasma.

**Results:**

After BCCAO/R, BCP prevented the increase of lipoperoxides occurring in the vehicle-treated rats in both cerebral cortex and plasma. In the frontal cortex, BCP further prevented activation of the endocannabinoid system (ECS), spared the docosahexaenoic acid (DHA), appeared to prevent the increase of cyclooxygenase-2 and increased the peroxisome-proliferator activated receptor-alpha (PPAR-alpha) protein levels, while, in plasma, BCP induced the reduction of arachidonoylethanolamide (AEA) levels as compared to vehicle-treated rats.

**Conclusions:**

Collectively, the pre-treatment with BCP, likely acting as agonist for CB2 and PPAR-alpha receptors, modulates in a beneficial way the ECS activation and the lipoperoxidation, taken as indicative of oxidative stress. Furthermore, our results support the evidence that BCP may be used as a dietary supplement to control the physiological response to the hypoperfusion/reperfusion-induced oxidative stress.

## Background

Several previous studies investigated on the effect of the transient Bilateral Common Carotid Artery Occlusion followed by Reperfusion (BCCAO/R) as a model of transient global hypoperfusion [1] that causes detectable and consistent molecular tissue changes, such as increase of the lipid peroxidation product malondialdehyde and superoxide dismutase activity [2], decrease of docosahexaenoic acid (DHA) and increase of oxidative stress and neuroinflammation markers [3, 4], and activation of the endocannabinoid system (ECS) [4]. The endocannabinoids (eCBs) are endogenous lipid mediators involved in a variety of biological processes spanning from neuromodulation to neuroprotection. The eCBs that have been studied the most are arachidonoylethanolamide (AEA or anandamide), belonging to the superfamily of N-acylethanolamines (NAEs), and 2-arachidonoylglycerol (2-AG). AEA and 2-AG are not stored in vesicles but they are synthesized ‘on demand’ and released from the N-acylphosphatidylethanolamines of the neuronal membrane bilayer immediately after their production when cells are challenged with potentially harmful stimuli [5–7]. Experimental evidence shows that eCBs, with their signaling-mediating receptors, and synthesizing/degrading enzymes, comprise an integrated extended system that is modulated by different and concurring molecular pathways [8, 9]. Experimental and clinical evidence supports a role for eCBs and related molecules in the preservation of metabolic homeostasis and in the regulation of brain response to oxidative stress. In particular, eCBs are involved in inflammation and act as endogenous neuroprotectants in cerebral ischemia [5, 7, 9–18]. Previous research has shown that 2-AG and AEA are substrates for COX-2 [19] and that the eCB neuroprotective activity may be mediated by preventing excessive expression of COX-2 [11, 20, 21]. Conversely, COX-2 is a key regulator of eCB signalling [22]. Moreover, it has been shown that the peroxisome-proliferator activated receptor (PPAR)-alpha mediates rapid effects of the anandamide congeners palmitoylethanolamide (PEA) and oleoylethanolamide (OEA) that behave as endogenous agonists at this receptor [23, 24].

We have previously reported on the beneficial effect of the in vivo administration of dietary *Pistacia lentiscus* L essential oils (E.O.) on the tissue physiological response to the BCCAO/R metabolic challenge [3]. In agreement with analyses of E.O. available in the literature, the E.O. showed a composition with relatively high concentration of terpenes and sesquiterpenes [3]. For some of these components a potent anti-inflammatory activity has been reported in different experimental rodent models [25, 26]. Unfortunately, the extension of those observations has been hampered both by the limited obtainable amount of E.O. and by the actual difficulty to extract from different plants an E.O. bearing exactly the same characteristics. In fact, the consistency of E.O. composition is markedly affected by biochemical adjustments that usually aid the plant to cope with environmental variations [27, 28]. Therefore, with the aim to extend our previous observations on the effect of natural compounds on the cerebral insult caused by a transient hypoperfusion/reperfusion, in this study we evaluated one of the E.*O. major* components [see 3], the beta-caryophyllene (BCP). BCP, a sesquiterpene found as a common constituent of the essential oils of numerous food plants [see 29] and primary component in *Cannabis sativa* L. [29], is a dietary phytocannabinoid acting as selective agonist for CB2 receptor [30, 31] and peroxisome-proliferator activating receptor alpha (PPAR-alpha) [32]. BCP displays many phytotherapeutic properties [33], including a marked anti-inflammatory activity that has been thoroughly demonstrated in different animal models of pain, such as carrageenan- and prostaglandin E1-induced edema [30, 34], formalin test [35], arthritis [36], colitis [37] and cisplatin-induced nephrotoxicity [38], in focal cerebral ischemia [39] and in vitro neurovascular unit against oxygen-glucose deprivation and re-oxygenation-induced injury [40].

In this study, we used the BCCAO/R model of hypoperfusion/reperfusion in the rat to probe the preventive effect of a single acute dose of BCP. With this aim, by means of HPLC, Western blot, and immunohistochemical analyses, we examined the concentrations of molecules involved in neuroinflammation and indicative of oxidative stress, such as eCBs and eCB congeners, the receptors CB1, CB2 and PPAR-alpha, lipoperoxides and COX-2. Occurrence of selected markers was investigated in the frontal cortex, area that is patently affected by the BCCAO/R [4], the temporal-occipital cortex, area that supposedly is not influenced by the hypoperfusion/reperfusion [4], and the plasma. Results are discussed in view of the possible utility of the ECS components and lipoperoxides as early markers of an ongoing transient cerebral global hypoperfusion and of the possibility to use BCP as a dietary supplement to control the physiological response to the hypoperfusion/reperfusion-induced oxidative stress.

## Methods

### Animals and keeping

One week before the experiment set off, male Wistar rats (Harlan-Italy, Udine, Italy), weighing 210 ± 20 g (mean ± SD) were housed under controlled temperature (21 ± 2 °C), relative humidity (60 ± 5%) and artificial 12 h light/dark cycle, avoiding all stressful stimuli. Animal handling and care throughout the experimental procedures met with national (Legislative Decree n. 26, 04/04/2014) and international (Directive 2010/63/EU in Europe) laws and policies. The experimental protocols were carried out in compliance with the guidelines of the Animal Ethics Committee of the University of Cagliari. Standard laboratory food (A04, Safe, Augy, France) and water were freely available ad libitum.

According to the optimum standard for the evaluation of lipids in tissue and plasma [41, 42], animals received no food for 12 h before surgery.

Rats (*n* = 112) were randomly assigned to 2 groups that received a pre-treatment 6 h before the surgery: one group (vehicle-treated; *n* = 60) was pre-treated with the vehicle, i.e. 0.3 ml of sunflower oil, while the other group (BCP-treated; *n* = 52) was previously gavage-fed (with the help of feeding needle) with 40 mg of BCP (Sigma-Aldrich, St Louis, Mo, USA) (corresponding to 180 mg/kg), dissolved in 0.3 ml of sunflower oil. Each group was further subdivided into sham-operated rats- represented by animals that underwent surgery without common carotid arteries (CCA) occlusion- and rats submitted to BCCAO/R and processed for lipid analysis in brain tissue and plasma (vehicle-treated *n* = 24; BCP-treated *n* = 20), western blot (vehicle-treated n = 24; BCP-treated n = 20) in brain tissue homogenates, and immunohistochemistry (vehicle-treated *n* = 12; BCP-treated n = 12) in brain tissue sections.

### Surgery

Surgical procedure for induction of BCCAO/R was adapted from the method of Iwasaki et al. [43] and performed in all cases between 13:00 and 16:30 p.m.. Rats were anesthetized with intraperitoneal administration of Equitesin (4.2% *w*/*v* chloral hydrate, 2.12% w/v MgSO4, 16.2% *w*/w pentobarbital, 39.6% w/w propylene glycol, and 10% w/w ethanol in sterile distilled H_2_O) (5 ml/100 g bodyweight). After a midline cervical incision and blunt dissection of muscles, the CCA were exposed caring to leave the vagus nerve intact. Cerebral blood flow reduction was produced by placement of two atraumatic microvascular clips for 30 min on CCA. The reperfusion period was achieved by removing the clips and restoring blood flow through the stenosed vessels for 60 min. The control animals, used to determine the effects of anaesthesia and surgical manipulation on the results, were represented by sham-operated rats.

### Sampling

At the end of the procedure, brain samples were collected either as fresh tissue for lipid analysis and Western blot or after transcardial perfusion fixation with ice cold 0.1 M phosphate buffer (PB), pH 7.4 for immunohistochemistry. The frontal cortex was rapidly dissected out by a transverse cut made at the level of the optic chiasm, at the approximate bregma level of − 1.0 mm [44], and frozen at − 80 °C until HPLC or western blot analysis. Temporal-occipital cortex, dissected out by a transverse cut at the approximate bregma level of − 4.5 mm, was also sampled as a control cortical area not irrorated by the internal carotid artery branches. Blood was quickly collected from the trunk of killed animals into heparinised tubes and centrifuged at 1500 g for 10 min at 8 °C. The resulting plasma was frozen at − 20 C° until assayed for lipids. Perfused brains were dissected out, fixed by overnight immersion in 4% formaldehyde in 0.1 M phosphate buffer (PB), pH 7.4 and then rinsed in 0.1 M PB, pH 7.3, containing 20% sucrose. After sucrose infiltration, samples were embedded in Optimal Cutting Temperature (OCT) medium for cryostat sectioning. For each assay, the investigator was blind with respect to the experimental condition of rats.

### Endocannabinoid and congener quantification

**Fig. 1:**
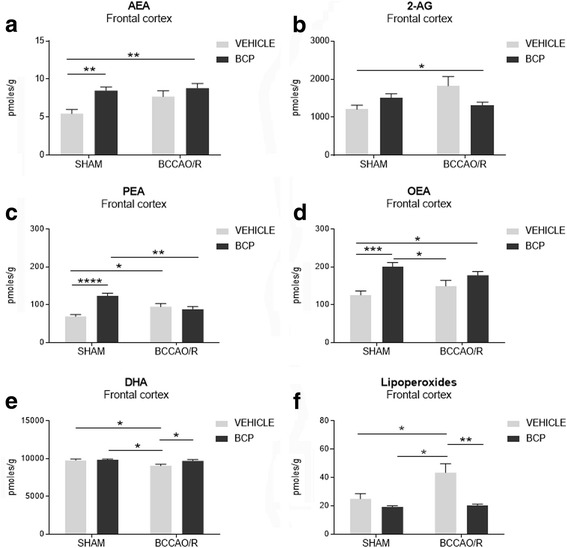
High performance liquid chromatography (HPLC) and liquid chromatography–atmospheric pressure chemical ionization–mass spectrometry analysis of frontal cortex (**a-f**) of sham-operated and bilateral common carotid artery occlusion followed by reperfusion (BCCAO/R) vehicle and BCP pre-treated rats. **a** 2-arachidonoylglycerol (2-AG), (**b**) arachidonoylethanolamide (AEA), (**c**) palmitoylethanolamide (PEA), (**d**) oleoylethanolamide (OEA), (**e**) docosahexaenoic acid (DHA) and (**f**) lipoperoxides concentrations are reported as mean values of 12 sham vehicle, 12 BCCAO/R vehicle, and 10 sham BCP pre-treated and 10 BCCAO/R BCP-treated rats. Error bars depict the standard error of mean (S.E.M.). Asterisks denote significant differences. Two-way ANOVA with the Bonferroni’s test for post-hoc analyses was applied to evaluate statistical differences between groups. *P* values < 0.05 were considered significant (refer to Table 1 for F and *p* values relevant to effects of BCCAO/R and BCP treatment and to interaction between them)

**Fig. 2:**
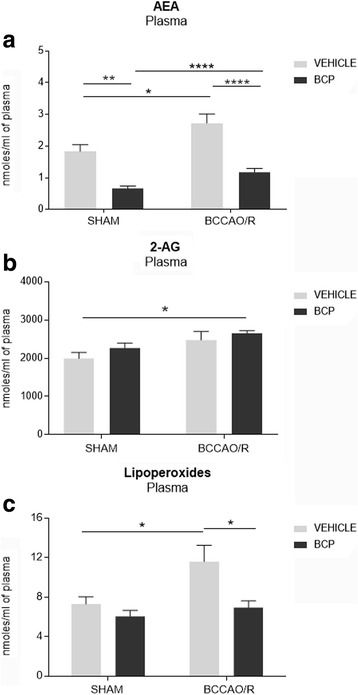
High performance liquid chromatography (HPLC) and liquid chromatography–atmospheric pressure chemical ionization–mass spectrometry analysis of plasma of sham-operated and bilateral common carotid artery occlusion followed by reperfusion (BCCAO/R) vehicle and BCP pre-treated rats. **a** Arachidonoylethanolamide (AEA), (**b**) 2-arachidonoylglycerol and (**c**) lipoperoxide concentrations are reported as mean values of 12 vehicle -either sham or BCCAO/R- rats and 10 BCP pre-treated -either sham or BCCAO/R- rats. Error bars depict the standard error of mean (S.E.M.). Asterisks denote significant differences. Two-way ANOVA with the Bonferroni’s test for post-hoc analyses was applied to evaluate statistical differences between groups. *P* values < 0.05 were considered significant (refer to Table 1 for F and p values relevant to effects of BCCAO/R and BCP treatment and to interaction between them)

Frozen tissues were homogenized and extracted with 50 mM chloroform/methanol/Tris-HCl, pH 7.5 (2:1:1, *v*/v), containing internal deuterated standards for AEA, 2-AG, PEA and OEA quantification by isotope dilution ([2H]^8^ AEA, [2H]^5^ 2-AG, [2H]^4^ PEA, [2H]^4^ OEA; Cayman Chemical, Ann Arbor, MI, USA). AEA, 2-AG, PEA, and OEA were quantified by liquid chromatography–atmospheric pressure chemical ionization–mass spectrometry [1100 HPLC system (Agilent Technologies, Santa Clara, CA, USA) equipped with MS Detector 6110 single quadrupole] and using selected ion monitoring at M1 values for the four compounds and their deuterated homologs, as described previously [41]. Concentrations (nmoles/g; nmoles/ml) are shown as histograms in Figs 1, 2.

### Measurement of fatty acid composition of tissue phospholipids

Total lipids were extracted from different brain areas using chloroform/methanol 2:1 (v/v). Aliquots were mildly saponified as previously described [45] in order to obtain free fatty acids for high-performance liquid chromatography (HPLC) analysis. Separation of fatty acids was carried out with an Agilent 1100 HPLC system (Agilent Technologies) equipped with a diode array detector as previously reported [42]. Concentrations (nmoles/g; nmoles/ml) are shown as histograms in Figs 1, 2.

### Western blot

**Fig. 3:**
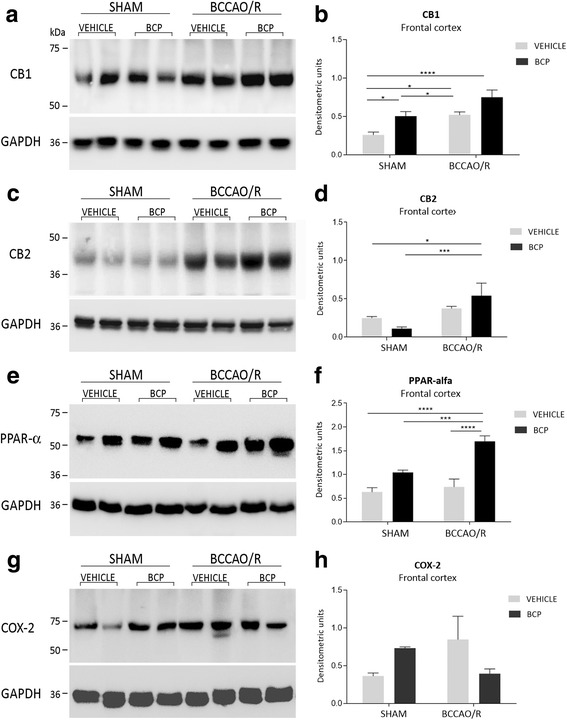
Western blot analysis of CB1 (**a, b**), CB2 (**c, d**), peroxisome-proliferator activated receptor-alpha (PPAR-alpha) (**e, f**) and cyclooxygenase-2 (COX-2) (**g, h**) in the frontal cortex of sham and bilateral common carotid artery occlusion followed by reperfusion (BCCAO/R) vehicle and BCP-pre-treated rats. **b, d**: densitometric analysis of the band gray levels expressed as a percentage of the optical density (O.D.) ratio of bands immunostained for CB1, CB2, PPAR-alpha and COX-2 to those of GAPDH. Data are reported as mean values of 12 sham-operated+vehicle, 12 BCCAO/R+ vehicle rats, 10 BCP-pre-treated sham-operated and 10 BCP-pre-treated BCCAO/R rats. Error bars depict the standard error of mean (S.E.M.). Asterisks denote significant differences. Two-way ANOVA with the Bonferroni’s test for post-hoc analyses was applied to evaluate statistical differences between groups. *P* values < 0.05 were considered significant (refer to Table 1 for F and p values relevant to effects of BCCAO/R and BCP treatment and to interaction between them)

Tissue homogenate were prepared in a 2% solution of sodium dodecyl sulfate (SDS) containing a cocktail of protease inhibitors (cOmplete, Mini Protease Inhibitor Cocktail Tablets, Roche, Basel, Switzerland). Protein concentrations were determined using the Lowry method of protein assay [46] with bovine serum albumin as standard. Proteins for each tissue homogenate (40 μg), diluted 3:1 in 4X loading buffer (NuPAGE LDS Sample Buffer 4X, Novex by Life Technologies, Carlsbad, CA, USA), were heated to 95 °C for 7 min and separated by SDS-polyacrilamide gel electrophoresis (SDS-PAGE) using precast polyacrylamide gradient gel (NuPAGE 4–12% Bis-Tris Gel Midi, Novex by Life Technologies) in the XCell4 Sure Lock™ Midi-Cell chamber (Life Technologies). Internal mw standards (Precision Plus Protein™ WesternC™ Standards, Bio-Rad, Hercules, CA, USA) were run in parallel. Two gels at a time were run for Coomassie staining and immunoblotting, respectively. Proteins for immunoblotting were electrophoretically transferred on a polyvinylidene fluoride membrane (Amersham Hybond™-P, GE Healthcare, Little Chalfont, United Kingdom) using the Criterion™ Blotter (Bio-Rad). Blots were blocked by immersion in 20 mM Tris base and 137 mM sodium chloride (TBS) containing 5% milk powder and 0.1% Tween 20 (TBS-T), for 60 min at room temperature and incubated overnight at 4 °C with rabbit polyclonal antisera directed against CB1 receptor (Synaptic System, Göttingen, Germany), diluted 1:500, CB2 receptor (Cayman Chemical, Ann Arbor, Mi, USA), diluted 1:1000, PPAR-α (Thermo Scientific, Waltham, MA, USA), diluted 1:1000, and COX-2 (residues 570–598) (Cayman Chemical), diluted 1:200 in TBS containing 5% milk powder and 0.02% sodium azide, used as primary antisera. After TBS-T rinse, blots were incubated for 60 min, at room temperature, with peroxidase-conjugated goat anti-rabbit serum (Sigma Aldrich), diluted 1:10,000 in TBS-T. Loading controls were obtained by stripping and immunostaining the membranes with a mouse monoclonal antibody against the housekeeping protein glyceraldehyde 3-phosphate dehydrogenase (GAPDH) (EMD Millipore, Darmstadt, Germany), diluted 1:1000, as primary antiserum, and a peroxidase-conjugated goat anti-mouse serum (EMD Millipore), diluted 1:5000, as secondary antiserum. In order to control for non specific staining, blots were stripped and incubated with the relevant secondary antiserum. After TBS-T rinse, protein bands were visualized using the ECL chemiluminescent system according to the protocol provided by the company (GE Healthcare), under ImageQuant LAS 4000. Approximate molecular weight (mw) and relative optical density (O.D.) of immunolabelled protein bands were evaluated by a “blind” examiner, and were quantified by comparing the position of relevant bands on the digital images with those of the GAPDH bands, respectively. The ratio of the intensity of CB1-, CB2-, PPAR-α- and COX-2-positive bands to the intensity of GAPDH-positive ones was used to compare relative expression levels of these proteins following BCCAO/R procedure. The O.D. was quantified by Image Studio Lite Software (Li-Cor, http: //www.licor.com/bio/products/software/image_studio_lite/) and is shown as histograms in Fig. 3.

### Immunohistochemistry

The avidin–biotin–peroxidase complex (ABC) technique was used to process cryostat semiconsecutive sections (16 μm thick), collected on chrome alum-gelatin coated slides. Coronal serial sections at + 4.70 to - 1.70 mm and at − 3.5 to - 8.0 mm Bregma levels, respectively, were used to focus the observations to frontal and temporal-occipital cortex (Paxinos and Watson, 2007). The endogenous peroxidase activity was blocked with 0.1% phenylhydrazine in phosphate buffered saline (PBS) containing 0.2% Triton X-100 (PBS/T) followed by incubation with 20% of either normal goat or normal horse serum (Vector, Burlingame, CA, USA) for 1 h at RT and then incubated with rabbit polyclonal antibody against CB1 (Synaptic System, Germany), diluted 1:1000, and against COX-2 (Cayman Chem., USA), diluted 1:300, as primary antibodies. Incubations with primary antiserum were carried out overnight at 4 °C. Biotin-conjugated goat anti-rabbit serum (Vector, Burlingame, CA, USA), diluted 1:400, was used as secondary antiserum. The ABC (BioSpa Div. Milan, Italy), diluted 1:250, followed by a solution of 0.1 M PB, pH 7.3, containing 0.05% 3,3′-diaminobenzidine (Sigma, Milan, Italy), 0.01% hydrogen peroxide and 0.04% nickel ammonium sulfate was used to reveal the reaction product. Incubations with secondary antiserum and ABC lasted 60 min and were performed at RT. Negative control preparations were obtained by incubating tissue sections in parallel with either PBS-T alone or with the relevant primary antiserum preabsorbed with an excess of the corresponding peptide antigen. Alternate sections were stained with modified Mayer’s hematoxylin. Slides were examined by the same examiner blinded to animals’ treatment with an Olympus BX61 microscope and digital images were captured with a Leica DF 450C camera.

### Statistical analysis

HPLC and Western blot data from the four experimental subgroups, i.e. vehicle- and BCP-treated sham-operated animals, and vehicle- and BCP-treated BCCAO/R rats, have been analyzed by two way analysis of variance (ANOVA) [main factors: a) BCP-treatment (i.e. vehicle- vs BCP-treatment) and b) BCCAO/R (i.e. sham-operation vs BCCAO/R] by using GraphPad Prism 7.03 for Windows (GraphPad Software, La Jolla California USA). Wherever appropriate (i.e. p for the main factors and their interaction < 0.05), multiple pair-wise contrasts were made and multiplicity adjusted *p* value for each comparison was calculated using the Bonferroni’s post hoc test.

## Results

### Analysis of eCB and fatty acid profiles in brain tissue

The effects of the administration of BCP on the concentrations of eCB, anandamide congeners and fatty acid profiles are reported in Table 1 and graphically shown in Fig. 1. Analysis of the tissue lipid extracts demonstrated that molecular changes were observed only in the frontal cortex. BCP- vs vehicle-treated differences were detected in the basal concentrations (i.e. in the sham-operated) of the ethanolamides. Thus, in pair-wise comparison of sham+BCP vs sham+vehicle rats, AEA, PEA and OEA were increased by 56% (post hoc *p* = 0.0094), 80% (post hoc adjusted *p* = < 0.0001) and 60% (post hoc adjusted *p* = 0.0005), respectively (Table 1; Fig. 1 a, c, d). Pair-wise comparisons with the Bonferroni’s test revealed that differences also occurred in both groups of BCCAO/R rats; thus, in BCCAO/R-BCP- vs BCCAO/R-vehicle-treated animals the concentration of DHA increased (+ 7%; post hoc adjusted *p* = 0.0447) (Table 1; Fig. 1e) while that of lipoperoxides decreased (− 53%; post hoc adjusted *p* = 0.0015) (Table 1; Fig. 1f). It appears from Fig. 1 that the BCP-pre-treatment reverts and/or reduces the molecular changes occurring after BCCAO/R. Accordingly, a significant BCP-treatment x BCCAO/R interaction was observed for 2-AG [F (1, 40): 6.427; *p* = 0.0153], PEA [F (1,40): 19.34, *p* <  0.0001] and lipoperoxides [F (], 39): 4.580; *p* = 0.0387] (Table 1; Fig. 1b–f). Importantly, the interaction between the ANOVA two main factors showed a tendency toward statistical significance in the case of OEA (*p* = 0.0615) and DHA (*p* = 0.0810) (Table 1). No statistically significant changes were observed in the temporal-occipital cortex (data not shown).

**Table 1:** F values and significance levels from two-way ANOVA performed on data obtained by means of HPLC-MS and Western blot in frontal cortex and plasma

			BCCAO/R		BCP treatment		BCP treatment x BCCAO/R		
		Marker	F value	p value	F value	p value	F value	p value	DF
*Frontal cortex*	HPLC-MS	AEA	4.152	0.0482	10.82	0.0021	2.279	ns	1, 40
		2-AG	1.804	ns	0.4065	ns	6.427	0.0153	1, 40
		PEA	0.4636	ns	12.29	0.0012	19.34	< 0.0001	1, 40
		OEA	0.0017	ns	18.19	0.0001	3.7	*0.0615*	1, 40
		DHA	6.972	0.0118	4.826	0.0339	3.205	*0.0810*	1, 40
		Lipoperoxides	5.665	0.0223	12.33	0.0011	4,580	0.0387	1, 39
	Western blot	CB1 receptor	16.83	0.0002	14.53	0.0004	0.0136	ns	1, 44
		CB2 receptor	15.05	0.0004	0.0396	ns	4.51	0.0401	1, 39
		PPAR-alpha	12.29	0.0011	39.66	< 0.0001	6.264	0.0163	1, 42
		COX-2	0.1578	ns	0.05464	ns	5.088	0.0299	1, 38
*Plasma*	HPLC-MS	AEA	11.78	0.0014	44.16	<0.0001	0.824	ns	1, 40
		2-AG	7.081	0.0112	1.907	ns	0.0916	ns	1, 40
		PEA	1.034	ns	4.605	0.0380	2.119	ns	1, 40
		OEA	0.7309	ns	2.321	0.1355	0.4911	ns	1, 40
		DHA	1.034	ns	4.605	0.0380	2.119	ns	1, 40
		Lipoperoxides	5.879	0.0199	7.68	0.0084	2.547	ns	1, 40

### eCB and fatty acid profiles in plasma

BCP- vs vehicle-treated differences were detected in the control animals for the AEA (− 64%; post hoc *p* <  0.0013), and in pair-wise contrasts of BCCAO/R-BCP-treated vs BCCAO/R-vehicle-treated rats, that showed a highly significant decrease of the AEA (− 56%; post hoc adjusted *p* <  0.0001) and lipoperoxides (− 38%; post hoc adjusted *p* = 0.0219) concentrations after BCP-treatment (Table 1; Fig. 2).

### Western blot

The effects of the administration of BCP on the concentrations of CB1, CB2, PPAR-alpha and COX-2 are reported in Table 1 and graphically shown in Fig. 3. As in the case of lipids, WB analysis of the tissue homogenates demonstrated that molecular changes were observed only in the frontal cortex. The effect of BCP-pre-treatment was evident in the pair-wise comparison of the sham+BCP vs sham+vehicle rats, with increase in the relative protein levels of CB1 (+ 53%; post hoc adjusted p 0.0481), and in the pair-wise contrast between the BCCAO/R rats, where PPAR-alpha levels significantly increased in BCCAO/R-BCP- vs BCCAO/R-vehicle-treated animals (+ 130%; post hoc adjusted p <  0.0001) (Table 1; Fig. 3). The post hoc analysis also showed an effect of the BCCAO/R per se; thus, relative protein levels increased in BCCAO/R- vs sham-vehicle-treated rats in the case of CB1 (+ 101%; post hoc adjusted *p* = 0.0279) and increased in BCCAO/R + BCP vs sham+BCP animals in the case of CB1 (+ 100%; post hoc adjusted *p* = 0.0433), CB2 (− 50%; post hoc adjusted *p* = 0.0010), and PPAR-alpha (+ 64%; post hoc adjusted *p* < 0.0001) (Table 1; Fig. 3). Consistently, BCP treatment x BCCAO/R interaction was observed for CB2 (*p* = 0.0401) and PPAR-alpha (*p* = 0.0163) (Table 1). As for COX-2, pair-wise comparisons showed no significant differences, however an interaction between the two ANOVA main factors was observed (*p* = 0.0299) (Table 1). No statistically significant changes were observed in the temporal-occipital cortex (data not shown).

The antibodies against CB1 and COX-2 were the only ones to produce a reliable immunostaining in tissue sections of rat cerebral cortex. For this reason, the following immunohistochemical data are based exclusively on the immunoreactivity obtained with them.

### Immunohistochemistry

**Fig. 4:**
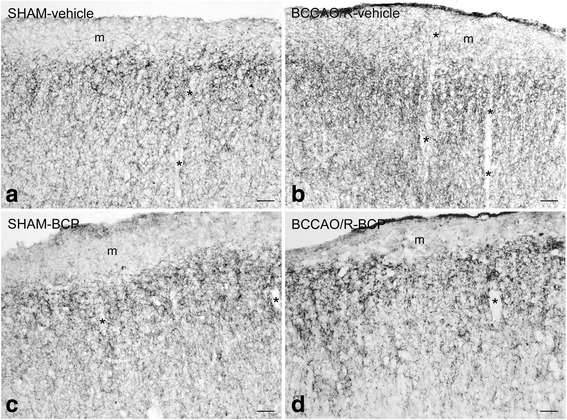
CB1-like immunoreactivity in frontal cortex coronal sections of sham-operated and bilateral common carotid artery occlusion followed by reperfusion (BCCAO/R) rats pre-treated with either the vehicle alone (**a, b**) or beta-caryophyllene (BCP) (**c, d**). Panels are representative of observations carried out in 6 rats for each group. Asterisks (*) point to blood vessels. m, molecular layer. Scale bars: 50 μm

**Fig. 5:**
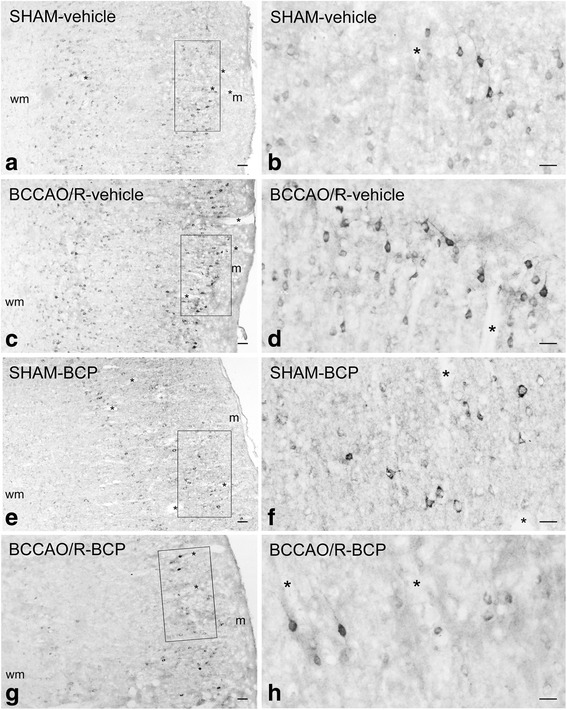
Cyclooxygenase-2 (COX-2)-like immunoreactivity in frontal cortex coronal sections of sham-operated and bilateral common carotid artery occlusion followed by reperfusion (BCCAO/R) rats pre-treated with either the vehicle alone (**a**-**d**) or betacaryophyllene (BCP) (**e**-**h**). Positive cell bodies are mostly distributed in the superficial cortical layers. **b**, **d**, **f**, **h**: detail at higher magnification of the microscopic field squared in **a**, **c**, **e**, **g**, respectively. Panels are representative of observations carried out in 6 rats for each group. Asterisks (*) point to blood vessels. m, molecular layer; wm; white matter. Scale bars: A, C, E, G = 50 μm; B, D, F, H = 25 μm

In order to associate the molecular changes observed by HPLC and western blot analyses and the tissue morphology, immunoreactivities to CB1 and COX-2 were also examined in the cerebral cortex (Figs. 4, 5). All markers labeled neuronal structures distributed throughout the rostro-caudal extension of the frontal cortex (Figs. 4, 5) and the temporal-occipital cortex (data not shown).

The CB1 receptor-antibody labelled a dense to moderate plexus of beaded fibers and some neuronal cell bodies distributed throughout the cortical layers in both vehicle- and BCP-treated rats (Fig. 4). As a general rule the density of labelled nerve terminals and fibers appeared higher in BCCAO/R animals (Fig. 4b, d) than in the sham-operated ones (Fig. 4a, c).

The antibody against COX-2 labelled mainly neuronal perikarya, that showed different density of intracytoplasmic immunostaining, and proximal neuronal processes (Fig. 5). Immunolabelled neurons were frequent in cortical layers II/III and V. As a general rule, vehicle-pre-treated rat brains showed that the staining intensity and density of labelled structures were higher in BCCAO/R (Fig. 5c, d) than in sham-operated rat brains (Fig. 5 a, b), whereas this difference was not evident in the BCP-pre-treated rats (Fig. 5 e-h).

## Discussion

The primary finding of this study is that the BCP treatment in a single acute dose exerts significant preventive effects against the tissue and plasmatic molecular changes triggered by the BCCAO/R, i.e. a) modulates the activation of the ECS by increasing basal tissue levels of 2-AG, AEA, PEA, OEA and relative protein levels of CB1 and CB2; b) decreases plasmatic levels of AEA in both sham and BCCAO/R rats; c) spares basal tissue levels of DHA after the BCCAO/R; e) induces a general increase of PPAR-alpha in BCCAO/R conditions; d) reverses the increase of lipoperoxide concentration following the BCCAO/R.

Data obtained match our previous observations on the outcome of the BCCAO/R with the same hypoperfusion and reperfusion durations used in this study [4], and further support the evidence that cerebral hypoperfusion/reperfusion induced by the BCCAO/R procedure, rather than producing a perceptible ischemic insult in the rodent brain, due to the collateral vessels that allow for a prompt cerebral blood flow compensation [47], triggers a series of metabolic changes that are precursor of oxidative stress and neuroinflammation [3, 4, 48, 49].

BCP, with its strong anti-inflammatory action, shows an extremely low toxicity in vivo (rat acute oral LD50 > 5000 mg/kg body weight) [50] and refs therein. The pharmacokinetics of its oral administration has been estimated for rats [51] and indicates that after a single dose of 50 mg/kg BCP reaches the mean maximum plasma concentration of 0.59 μM. Moreover, BCP bio-availability may persist for hours since the maximum concentration and the terminal elimination half-life are reached in 3.50 h and 4.07 h, respectively [51].

### BCP effects on the eCB system following hypoperfusion/reperfusion

Our results, demonstrating that a single dose of BCP may modulate lipid metabolism and related receptor proteins, are in agreement with pharmacokinetic data [51] and with data on the effectiveness of BCP-pretreatment and extend our previous observations on the beneficial effects of the *Pistacia lentiscus* L. essential oil in slightly different experimental conditions [3].

Several BCP properties that have been recently reported can explain the molecular changes we observed in the rat BCCAO/R model [20, 30, 31, 34, 35, 37, 38, 52–54]. Indeed, BCP is a selective agonist for CB2 and PPAR-alpha receptors [30–32, 55] and, in addition, has been shown to inhibit fatty acid amide hydrolase (FAAH) [20, 31, 54] and COX-2 [52].

It is known that brain ischemia and reperfusion activate the ECS, by driving a combination of biochemical adaptations of the NAE degrading and synthesizing enzymes that, collectively, lead to the accumulation of eCBs [12, 56]. Similarly, the BCCAO/R model of transient global hypoperfusion/reperfusion activates the ECS [present data; 4]. Among the eCB congeners, PEA represents an important player of the endogenous defense against neuroinflammation [11, 57–59]. Importantly, beyond the modulation of the immune cells [59], PEA exerts its neuroprotective effects by activating PPAR-alpha [23, 24] and blunting COX-2 activity [11]. Thus, exogenous administration of PEA in an acute stroke model is effective in reducing the infarct size [7]. Moreover, in stroke patients eCBs and congeners, including PEA, have been shown to increase during the acute ischemic phase during which they may play a role through multiple potential mechanisms [17]. Our data indicate that, after a single dose of BCP, the tissue levels of PEA, as well as levels of AEA and OEA, increase in the sham-operated, while, as a general rule, they do not change after induction of BCCAO/R.

Though it is difficult to speculate on the possible biological significance of the increase of AEA, PEA and OEA levels in basal conditions, it can be suggested a scenario where BCP, known to elicit a full agonist action on CB2 receptors and PPAR-alpha and gamma isoforms [32, 55], interferes with the endogenous signaling of eCB and congeners by adjusting their relative levels in both basal conditions and after BCCAO/R. Interestingly, it has been shown that PPAR-alpha induction elicits an increase of PEA and OEA [60]. Thus, in the presence of exogenous BCP, levels of AEA, 2-AG and PEA in the BCCAO/R rat frontal cortex are either unchanged or reduced; these findings, together with the increases of CB receptors and PPAR-alpha and the marked decrease of lipoperoxides, and the interaction between BCCAO/R and BCP-treatment observed for the COX-2 expression lead to suggest that a modulation of the endogenous anti-inflammatory milieu is occurring in the tissue challenged by the BCCAO/R-induced oxidative stress [see 23]. In our basic experimental setting it is not feasible to speculate whether the PPAR-alpha receptor is induced by its NAE ligands, including PEA, or viceversa. However, it has been suggested that enhancing the tissue availability of PEA through inhibition or modulation of its enzymatic breakdown may represent a complementary therapeutic approach to counteract neuroinflammation [20, 56, 60]. Thus, it is tempting to speculate that BCP may increase the eCB and congeners by activating PPAR-alpha [32, 55, 61], and initiate a preventive action on some aspects of the tissue physiological response to the BCCAO/R-induced oxidative stress.

The present data are also consistent with previous results obtained in serum from patients undergoing carotid endoarterectomy, where increased levels of lipoperoxides and isoprostanes and a concomitant increase of their catabolism in peroxisomes have been shown to be directly correlated to the hypoperfusion/reperfusion-induced oxidative challenge [62]. In particular, it is interesting that peroxisomal beta-oxidation increased during the first 30 min of reperfusion only in patients having contralateral carotid stenosis higher than 50% [62]. On the other hand, recent polypharmacological studies have pointed out that, in a therapeutic setting, a critical role is played by the synergistic modulatory effects due to both the modulation of CB2 receptors and the increase of AEA levels as a consequence of the inhibition of its main degrading enzyme [31]. Thus, more specific experiments should be carried out to evaluate whether, upon specific inhibition of FAAH and/or combined inhibition of COX-2, the BCP effects are confined to brain.

Interestingly, our data further show that BCP decreases AEA levels in plasma. Though it is difficult to speculate whether the plasmatic decrease of AEA is related to the eCB modifications in brain tissue, it can be hypothesized that the general decrease of peripheral AEA observed in both the sham+BCP and the BCCAO/R + BCP animals may reflect the integrity of brain tissue. Clinical studies support the notion that neurological and neuropsychiatric disorders are characterized by detectable changes in eCB plasma levels and that the AEA and PEA plasma levels are correlated with neurological disability, so that patients with higher AEA and PEA levels had greater neurological impairment [17 and refs therein].

In this study, we have also demonstrated that, after BCCAO/R, higher concentration of eCBs are associated with an increase of the relative levels of CB1 and CB2 receptor proteins. Both these receptors may contribute to the physiological tissue response to the BCCAO/R challenge. As already proposed [63–65], a parallel raise in eCBs and CB1 receptors indicates a sensitization of the cannabinoidergic system that may contribute to regulate cellular functions that depend upon CB1 receptor activation. Thus, after BCCAO/R, this sensitization would be able to modulate events such as neurotransmitter release, calcium cellular influx, oxidative stress damage, and vascular tone that appear to be crucial in the response of cerebral tissue to the hypoperfusion/reperfusion insult. Interestingly, it has been reported that CB2 remains inactive under physiological conditions [39, 53] and that its activation is crucial in injury models [66, 67], such as middle cerebral artery occlusion (MCAO) and reperfusion [39, 53]. Thus the BCP pre-treatment enhances the biological activities that have been associated to CB2 ischemia/reperfusion-induced activation, such as amelioration of microcirculation dysfunction and anti-inflammation [39, 53]. The parallel increase of cortical CB2 relative protein levels may also contribute to the physiological response by regulating the production of pro-inflammatory molecules by glial cells, through which CB2 may either prevent the detrimental effects of neuroinflammatory reaction or participate in adaptive changes to the brain insult [53, 68, 69]. Further, in light of the evidence that eCBs may act as ligands for receptors other than CBs [70], it is interesting that the endocannabinoid and endovanilloid pathways have been found to antagonistically interact to adjust synaptic strength of inhibitory synapses [71].

As for OEA, BCP pre-treatment causes its positive modulation in sham animals suggesting a complex role for OEA in the BCAO/R-induced insult perhaps linked, as suggested above, to the reported BCP-induced activation of PPAR-alpha [32, 55]. In agreement with earlier experimental evidence arguing against the utility of OEA as neuroprotectant prior to ischemic stroke [72], it can be suggested that in our experimental setting the PPAR-alpha receptor is playing a dual role, both anti-inflammatory, likely via repression of NF-kB signalling [73], and anti-oxidative stress by enhancing lipoperoxide degradation [74].

### BCP effects on the hypoperfusion/reperfusion-induced oxidative stress

Consistently with our previous findings in absence of any medicinal treatment [3, 4], one of the unfavorable biological effects through which BCCAO/R can affect the tissue homeostasis is the decrease of DHA tissue content, a polyunsaturated fatty acid that is naturally abundant and avidly retained in the brain [75, 76]. Interestingly, the pre-treatment with BCP totally prevented the BCCAO/R-induced decrease of DHA. The disruption of DHA levels is a key factor that affects the efficiency of membrane-depending molecular mechanisms [77]. In fact, DHA is particularly predisposed to lipid peroxidation [76] and, therefore, potentially apt to contribute to the hypoperfusion/reperfusion-induced oxidative stress. The observation that BCP pre-treatment also induces an increase of PPAR-alpha relative levels, an inverse correlation between COX-2 relative levels in BCCAO/R-vehicle- vs BCCAO/R-BCP-pre-treated rats (as shown by the statistically significant BCP-pre-treatment*BCCAO/R interaction), and a decrease of lipoperoxide concentration, leads to suggest that BCP activates multiple and concurrent factors to counteract the BCCAO/R-induced tissue reaction, hence helping to preserve the brain structure. Interestingly, it has been recently reported that the exogenous administration of BCP in combination with DHA has a unique effect in inducing analgesia after formalin injection [78]. Since the synergic activity of BCP and DHA took place in an experiment carried out in vitro, in which the results could not be explained by systemic effects, Fiorenzani et al. [78] proposed that it is likely that both BCP and DHA show competitive binding/interaction for the same receptor. Indeed, there is evidence that DHA activates PPAR-alpha [79, 80], and also inhibits COX-2 and prostaglandins formation during neuroinflammation [81]. In this context, it is relevant that recent prophylactic and therapeutic approaches for cerebrovascular disease take into account the pathways of brain accretion and delivery of DHA [77, 82].

As a matter of fact, in the present study, BCP treatment triggers a marked decrease of tissue and plasmatic concentrations of lipoperoxides. Fatty acid hydroperoxides are quite unstable compounds capable of extending the free radical oxidative damage and form pro-inflammatory substances [83, 84]. Several physiopathological conditions, not necessarily associated to early obvious neurological signs [85], share the occurrence of cerebral hypoperfusion episodes for which the detection of molecular indicators in the early hours may be useful in clinical settings to prevent irreversible cerebral damage. Whether plasmatic changes of AEA and lipoperoxides could represent additional specific markers in humans and if BCP holds promise as an effective nutraceutical compound should be further investigated.

## Conclusions

The present study showed that the BCP pre-treatment has effects on the entire ECS and prevents the BCCAO/R-induced increase of lipoperoxides as well as the reduction of COX-2 relative protein level, thus contributing to avoid the onset of a pro-inflammatory milieu. These data support the concept that, at both tissue and peripheral levels, multiple mechanisms may cope with the molecular dysregulation induced by the BCCAO/R. BCP, with its complex bioactivity, has been approved by the FDA for use in food [30] and so far, it has been shown to have no genotoxic or cytotoxic effects in vivo [55, 86]. The BCP extremely low toxicity [48 and refs therein] and the pharmacokinetics of its oral administration, recently estimated for rats [49], indicate that it may be an excellent therapeutic agent to preserve the tissue metabolism and prevent the upshots of the hypoperfusion/reperfusion challenge.

## Abbreviations

2-AG: 2-arachidonoylglycerol
AEA: Arachidonoylethanolamide or anandamide
BCCAO/R: Bilateral Common Carotid Artery Occlusion followed by Reperfusion
BCP: Beta-caryophyllene
CB: Cannabinoid receptor
COX-2: Cyclooxygenase-2
DHA: Docosahexaenoic acid
E.O.: *Pistacia lentiscus* L essential oils
eCBs: Endocannabinoids
ECS: Endocannabinoid system
FAAH: Fatty acid amide hydrolase
NAEs: N-acylethanolamines
OEA: Oleoyl ethanolamide
PEA: Palmitoyl ethanolamide
PPAR-alpha: Peroxisome-proliferator activated receptor-alpha

## References

[1] Traystman RJ, Kirsch JR, Koehler RC (1991). Oxygen radical mechanisms of brain injury following ischemia and reperfusion. J Appl Physiol.

[2] Yanpallewar SU, Hota D, Rai S, Kumar M, Acharya SB (2004). Nimopidine attenuates biochemical, behavioral and histopathological alterations induced by acute transient and long-term bilateral common carotid occlusion in rats. Pharmacol Res.

[3] Quartu M, Serra MP, Boi M, Pillolla G, Melis T, Poddighe L, Del Fiacco M, Falconieri D, Carta G, Murru E, Cordeddu L, Piras A, Collu M, Banni S (2012). Effect of acute administration of Pistacia lentiscus L essential oil on rat cerebral cortex following transient bilateral common carotid artery occlusion. Lipids Health Dis.

[4] Quartu M, Poddighe L, Melis T, Serra MP, Boi M, Lisai S, Carta G, Murru E, Muredda L, Collu M, Banni S (2017). Involvement of the endocannabinoid system in the physiological response to transient common carotid artery occlusion and reperfusion. Lipids Health Dis.

[5] Schäbitz, W.R., Giuffrida, A., Berger, C., Aschoff, A., Schwaninger, M., Schwab, S., Piomelli, D. Release of fatty acid amides in a patient with hemispheric stroke: a microdialysis study. Stroke 2002, 33, 2112–2114. doi: 10.1161/01.STR.0000023491.63693.18. 10.1161/01.str.0000023491.63693.18 12154273

[6] Melis M, Pillolla G, Bisogno T, Minassi A, Petrosino S, Perra S, Muntoni AL, Lutz B, Gessa GL, Marsicano G, Di Marzo V, Pistis M (2006). Protective activation of the endocannabinoid system during ischemia in dopamine neurons. Neurobiol Dis.

[7] Schomacher M, Müller HD, Sommer C, Schwab S, Schäbitz WR (2008). Endocannabinoids mediate neuroprotection after transient focal cerebral ischemia. Brain Res.

[8] Di Marzo V, Petrosino S (2008). Endocannabinoids and the regulation of their levels in health and disease. Curr Opin Lipidol.

[9] Di Marzo V (2009). The endocannabinoid system: its general strategy of action, tools for its pharmacological manipulation and potential therapeutic exploitation. Pharmacol Res.

[10] Conti S, Costa B, Colleoni M, Parolaro D, Giagnoni G (2002). Antiinflammatory action of endocannabinoid palmitoylethanolamide and the synthetic cannabinoid nabilone in a model of acute inflammation in the rat. Br J Pharmacol.

[11] Costa B, Conti S, Giagnoni G, Colleoni M (2002). Therapeutic effect of the endogenous fatty acid amide, palmitoylethanolamide, in rat acute inflammation: inhibition of nitric oxide and cyclo-oxygenase systems. Br J Pharmacol.

[12] Hillard CJ (2008). Role of cannabinoids and endocannabinoids in cerebral ischemia. Curr. Pharm. Res..

[13] Centonze D, Battistini L, Maccarrone M (2008). The endocannabinoid system in peripheral lymphocytes as a mirror of neuroinflammatory diseases. Curr Pharm Res.

[14] Pacher P, Haskó G (2008). Endocannabinoids and cannabinoid receptors in ischaemia-reperfusion injury and preconditioning. Br J Pharmacol.

[15] Zhang M, Adler MW, Abood ME, Ganea D, Jallo J, Tuma RF (2009). CB2 receptor activation attenuates microcirculatory dysfunction during cerebral ischemic/reperfusion injury. Microvasc Res.

[16] Pellegrini-Giampietro DE, Mannaioni G, Bagetta G (2009). Post-ischemic brain damage: the endocannabinoid system in the mechanisms of neuronal death. FEBS J.

[17] Naccarato M, Pizzuti D, Petrosino S, Simonetto M, Ferigo L, Grandi FC, Pizzolato G, Di Marzo V (2010). Possible anandamide and palmitoylethanolamide involvement in human stroke. Lipids Health Dis.

[18] Shohami E, Cohen-Yeshurun A, Magid L, Algali M, Mechoulam R (2011). Endocannabinoids and traumatic brain injury. Br J Pharmacol.

[19] Kozak KR, Crews BC, Morrow JD, Wang LH, Ma YH, Weinander R, Jakobsson PJ, Marnett LJ (2002). Metabolism of the endocannabinoids, 2-arachidonylglycerol and anandamide, into prostaglandin, thromboxane, and prostacyclin glycerol esters and ethanolamides. J Biol Chem.

[20] Jhaveri MD, Richardson D, Robinson I, Garle MJ, Patel A, Sun Y, Sagar DR, Bennett AJ, Alexander SP, Kendall DA, Barrett DA, Chapman V (2008). Inhibition of fatty acid amide hydrolase and cyclooxygenase-2 increases levels of endocannabinoid related molecules and produces analgesia via peroxisome proliferator-activated receptor-alpha in a model of inflammatory pain. Neuropharmacology.

[21] Zhang J, Chen C (2008). Endocannabinoid 2-arachidonoylglycerol protects neurons by limiting COX-2 elevation. J Biol Chem.

[22] Hermanson DJ, Gamble-George JC, Marnett LJ, Patel S (2014). Substrate-selective COX-2 inhibition as a novel strategy for therapeutic endocannabinoid augmentation. Trends Pharmacol Sci.

[23] Lo Verme J, Fu J, Astarita G, La Rana G, Russo R, Calignano A, Piomelli D (2005). The nuclear receptor peroxisome proliferator-activated receptor-alpha mediates the anti-inflammatory actions of palmitoylethanolamide. Mol Pharmacol.

[24] Hansen HH (2006). Palmitoylethanolamide and other anandamide congeners. Proposed role in the diseased brain. Exp Neurol.

[25] Tambe Y, Tsujiuchi H, Honda G, Ikeshiro Y, Tanaka S (1996). Gastric cytoprotection of the non-steroidal anti-inflammatory sesquiterpene, betacaryophyllene. Planta Med.

[26] Gertsch J (2011). Botanical drugs, synergy, and network pharmacology: forth and back to intelligent mixtures. Planta Med.

[27] Barra A, Coroneo V, Dessi S, Cabras P, Angioni A (2007). Characterization of the volatile constituents in the essential oil of *Pistacia lentiscus* L. from different origins and its antifungal and antioxidant activity. J Agric Food Chem.

[28] Chryssavgi G, Vassiliki P, Athanasios M, Kibouris T, Michael K (2008). Essential oil composition of *Pistacia lentiscus* L. and *Myrtus communis* L., evaluation of antioxidant capacity of methanolic extracts. Food Chem.

[29] Hendriks H, Malingre T, Battermann S, Boss R (1975). Mono- and sesquiterpene hydrocarbons of the essential oil of cannabis sativa. Phytochemistry.

[30] Gertsch J, Leonti M, Raduner S, Racz I, Chen JZ, Xie XQ, Altmann KH, Karsak M, Zimmer A (2008). Beta-caryophyllene is a dietary cannabinoid. Proc Natl Acad Sci U S A.

[31] Chicca A, Caprioglio D, Minassi A, Petrucci V, Appendino G, Taglialatela-Scafati O, Gertsch J (2014). Functionalization of β-caryophyllene generates novel polypharmacology in the endocannabinoid system. ACS Chem Biol.

[32] Wu C, Jia Y, Hae Lee J, Jun H, Lee H-S, Hwang K-Y, Lee S-J (2014). Bioorganic & Medicinal Chemistry Letters.

[33] Russo EB (2011). Taming THC: potential cannabis synergy and phytocannabinoid-terpenoid entourage effects. Br J Pharmacol.

[34] Fernandes ES, Passos GF, Medeiros R, da Cunha FM, Ferreira J, Campos MM, Pianowski LF, Calixto JB (2007). Anti-inflammatory effects of compounds alpha-humulene and (−)-trans-caryophyllene isolated from the essential oil of Cordia Verbenacea. Eur J Pharmacol.

[35] Klauke, A.L., Racz, I., Pradier, B., Markert, A., Zimmer, A.M., Gertsch, J., Zimmer, A. The cannabinoid CB2 receptor-selective phytocannabinoid beta-caryophyllene exerts analgesic effects in mouse models of inflammatory and neuropathic pain. Eur Neuropsychopharmacol 2014, 24, 608–620. doi:10.1016/j.euroneuro.2013.10.008. 10.1016/j.euroneuro.2013.10.008 24210682

[36] Agarwal, R.B., Rangari, V.D. Phytochemical investigation and evaluation of anti-inflammatory and anti-arthritic activities of essential oil of Strobilanthus ixiocephala Benth. Indian J Exp Biol 2003, 41, 890–894. PMID: 15248491. 15248491

[37] Bento AF, Marcon R, Dutra RC, Claudino RF, Cola M, Leite DF, Calixto JB (2011). β-Caryophyllene inhibits dextran sulfate sodium-induced colitis in mice through CB2 receptor activation and PPARγ pathway. Am J Pathol.

[38] Horváth B, Mukhopadhyay P, Kechrid M, Patel V, Tanchian G, Wink DA, Gertsch J, Pacher P (2012). β-Caryophyllene ameliorates cisplatin-induced nephrotoxicity in a cannabinoid 2 receptor-dependent manner. Free Radic Biol Med.

[39] Chang HJ, Kim JM, Lee JC, Kim WK, Chun HS (2013). Protective effect of β-caryophyllene, a natural bicyclic sesquiterpene, against cerebral ischemic injury. J Med Food.

[40] Tian X, Peng J, Zhong J, Yang M, Pang J, Lou J, Li M, An R, Zhang Q, Xu L, Dong Z (2016). β-Caryophyllene protects in vitro neurovascular unit against oxygen-glucose deprivation and re-oxygenation-induced injury. J Neurochem.

[41] Piscitelli F, Carta G, Bisogno T, Murru E, Cordeddu L, Berge K, Tandy S, Cohn JS, Griinari M, Banni S, Di Marzo V (2011). Effect of dietary krill oil supplementation on the endocannabinoidome of metabolically relevant tissues from high-fat-fed mice. Nutr Metab.

[42] Melis, M.P., Angioni, E., Carta, G., Murru, E., Scanu, P., Spada, S., Banni, S. Characterization of conjugated linoleic acid and its metabolites by RPHPLC with diode array detector. Eur J Lipid Sci Technol 2001, 103: 617–621. DOI: 10.1002/1438-9312(200109)103:9<617::AID-EJLT6170>3.0.CO;2-C.

[43] Iwasaki Y, Ito S, Suzuki M, Nagahori T, Yamamoto T, Konno H (1989). Forebrain ischemia induced by temporary bilateral common carotid occlusion in normotensive rats. J Neurol Sci.

[44] Paxinos, G., Watson, C. The Rat Brain in Stereotaxic Coordinates. 2007, Sixth ed. academic press. Elsevier, Amsterdam-Boston Hardcover ISBN: 9780125476126.

[45] Banni S, Carta G, Contini MS, Angioni E, Deiana M, Dessi MA, Melis MP, Corongiu FP (1996). Characterization of conjugated diene fatty acids in milk, dairy products, and lamb tissues. J Nutr Biochem.

[46] Lowry OH, Rosebrough NJ, Farr AL, Randall RJ (1951). Protein measurements with the Folin phenol reagent. J. Biol. Chem.

[47] Coyle P, Panzenbeck MJ (1990). Collateral development after carotid artery occlusion in Fischer 344 rats. Stroke.

[48] Lapi D, Vagnani S, Pignataro G, Esposito E, Paterni M, Colantuoni A (2012). Protective effects of quercetin on rat pial microvascular changes during transient bilateral common carotid artery occlusion and reperfusion. Front Physiol.

[49] Lapi D, Vagnani S, Pignataro G, Esposito E, Paterni M (2012). Colantuoni. A Rat pial microvascular responses to transient bilateral common carotid artery occlusion and reperfusion: quercetin's mechanism of action Front Physiol.

[50] Kamikubo R, Kai K, Tsuji-Naito K, Akagawa M (2016). β-Caryophyllene attenuates palmitate-induced lipid accumulation through AMPK signaling by activating CB2 receptor in human HepG2 hepatocytes. Mol Nutr Food Res.

[51] Liu H, Yang G, Tang Y, Cao D, Qi T, Qi Y, Fan G (2013). Physicochemical characterization and pharmacokinetics evaluation of β013. Int J Pharm.

[52] Park KR, Nam D, Yun HM, Lee SG, Jang HJ, Sethi G, Cho SK, Ahn KS. β-Caryophyllene oxide inhibits growth and induces apoptosis through the suppression of PI3K/AKT/mTOR/S6K1 pathways and ROS-mediated MAPKs activation. Cancer Lett. 312:2011, 178–88. doi:10.1016/j.canlet.2011.08.001. 10.1016/j.canlet.2011.08.001 21924548

[53] Choi IY, Ju C, Anthony Jalin AM, Lee DI, Prather PL, Kim WK (2013). Activation of cannabinoid CB2 receptor-mediated AMPK/CREB pathway reduces cerebral ischemic injury. Am J Pathol.

[54] Kwilasz AJ, Abdullah RA, Poklis JL, Lichtman AH, Negus SS (2014). Effects of the fatty acid amide hydrolase (FAAH) inhibitor URB597 on pain-stimulated and pain-depressed behavior in rats. Behav Pharmacol.

[55] Sharma, C., Al Kaabi, J.M., Nurulain, S.M., Goyal, S.N., Kamal, M.A., Ojha, S. Polypharmacological properties and therapeutic potential of β-Caryophyllene: a dietary Phytocannabinoid of pharmaceutical promise. Curr Pharm Des 2016, 22, 3237–3264. DOI: 10.2174/1381612822666160311115226. 10.2174/1381612822666160311115226 26965491

[56] Skaper, S.D., Facci, L., Barbierato, M., Zusso, M., Bruschetta, G., Impellizzeri, D., Cuzzocrea, S., Giusti, P. N-Palmitoylethanolamine and neuroinflammation: a novel therapeutic strategy of resolution. Mol Neurobiol 2015, 52, 1034–1042. DOI: 10.1007/s12035-015-9253-8. 10.1007/s12035-015-9253-8 26055231

[57] De Filippis D, D'Amico A, Cipriano M, Petrosino S, Orlando P, Di Marzo V, Iuvone T (2010). Levels of endocannabinoids and palmitoylethanolamide and their pharmacological manipulation in chronic granulomatous inflammation in rats. Pharmacol Res.

[58] Balvers, M.G., Verhoeckx, K.C., Meijerink, J., Wortelboer, H.M., Witkamp, R.F. Measurement of palmitoylethanolamide and other N-acylethanolamines during physiological and pathological conditions. CNS Neurol Disord Drug Targets. 2013, 12, 23–33. URL: http://www.ncbi.nlm.nih.gov/pubmed/23394528. 10.2174/1871527311312010007 23394528

[59] Skaper SD, Facci L, Giusti P (2013). Glia and mast cells as targets for palmitoylethanolamide, an anti-inflammatory and neuroprotective lipid mediator. Mol Neurobiol.

[60] Turcotte C, Chouinard F, Lefebvre JS, Flamand N (2015). Regulation of inflammation by cannabinoids, the endocannabinoids 2-arachidonoyl-glycerol and arachidonoyl-ethanolamide, and their metabolites. J Leukoc Biol.

[61] Melis M, Carta G, Pistis M, Banni S (2013). Physiological role of peroxisome proliferator-activated receptors type α on dopamine systems. CNS Neurol Disord Drug Targets.

[62] Banni S, Montisci R, Sanfilippo R, Finco G, Sanna D, Giordano E, Murru E, Cordeddu L, Carta G, Banni D, Marchi A (2010). Physiological response to lipid peroxidation in ischemia and reperfusion during carotid endarterectomy. Lipids Health Dis.

[63] Fernández-Ruiz J, Moro MA, Martínez-Orgado J. Cannabinoids in Neurodegenerative Disorders and Stroke/brain trauma: from preclinical models to clinical applications. Neurotherapeutics. 2015; doi:10.1007/s13311-015-0381-7. 10.1007/s13311-015-0381-7 PMC4604192 26260390

[64] Svízenská I, Dubový P, Sulcová A (2008). Cannabinoid receptors 1 and 2 (CB1 and CB2), their distribution, ligands and functional involvement in nervous system structures-a short review. Pharmacol Biochem Behav.

[65] Vinod KY, Arango V, Xie S, Kassir SA, Mann JJ, Cooper TB, Hungund BL (2005). Elevated levels of endocannabinoids and CB1 receptor-mediated G-protein signalling in the prefrontal cortex of alcoholic suicide victims. Biol Psychiatry.

[66] Bisogno T, Oddi S, Piccoli A, Fazio D, Maccarrone M (2016). Type-2 cannabinoid receptors in neurodegeneration. Pharmacol Res.

[67] Javed H, Azimullah S, Haque ME, Ojha SK (2016). Cannabinoid Type 2 (CB2) receptors activation protects against oxidative stress and neuroinflammation associated dopaminergic neurodegeneration in rotenone model of parkinson’s disease. Front Neurosci.

[68] Parmentier-Batteur S, Jin K, Mao XO, Xie L, Greenberg DA (2002). Increased severity of stroke in CB1 cannabinoid receptor knock-out mice. J Neurosci.

[69] Benito C, Tolón RM, Pazos MR, Núñez E, Castillo AI, Romero J (2008). Cannabinoid CB2 receptors in human brain inflammation. Br J Pharmacol.

[70] Di Marzo V, Breivogel CS, Tao Q, Bridgen DT, Razdan RK, Zimmer AM, Zimmer A, Martin BRL (2000). Metabolism, and pharmacological activity of anandamide in CB(1) cannabinoid receptor knockout mice: evidence for non-CB(1), non-CB(2) receptor-mediated actions of anandamide in mouse brain. J Neurochem.

[71] Lee, S.H., Ledri, M., Tóth, B., Marchionni, I., Henstridge, C.M., Dudok, B., Kenesei, K., Barna, L., Szabó, S.I., Renkecz, T., Oberoi, M., Watanabe, M., Limoli, C.L., Horvai, G., Soltesz, I., Katona, I. Multiple forms of endocannabinoid and endovanilloid signaling regulate the tonic control of GABA release. J Neurosci 2015, 35,10039–10057. DOI: 10.1523/JNEUROSCI.4112-14.2015. 10.1523/JNEUROSCI.4112-14.2015 PMC4495235 26157003

[72] Sun Y, Alexander SP, Garle MJ, Gibson CL, Hewitt K, Murphy SP, Kendall DA, Bennett AJ (2007). Cannabinoid activation of PPAR alpha; a novel neuroprotective mechanism. Br J Pharmacol.

[73] Staels B, Koenig W, Habib A, Merval R, Lebret M, Torra IP, Delerive P, Fadel A, Chinetti G, Fruchart JC, Najib J, Maclouf J, Tedgui A (1998). Activation of human aortic smooth-muscle cells is inhibited by PPARα but not by PPARγ activators. Nature.

[74] Reddy JK, Hashimoto TP (2013). Beta-oxidation and peroxisome proliferator-activated receptor alpha: an adaptive metabolic system. Ann Rev Nutr.

[75] Garcia MC, Ward G, Ma YC, Salem N, Kim HVE (1998). Of docosahexaenoic acid on the synthesis of phosphatidylserine in rat brain in microsomes and c6 glioma cells. J Neurochem.

[76] Niemoller DT, Bazan NG (2010). Docosahexaenoic acid neurolipidomics. Prostaglandins Other Lipid Mediat.

[77] Mayurasakorn K, Williams JJ (2011). Ten, V.S., Deckelbaum, R.J. Docosahexaenoic acid: brain accretion and roles in neuroprotection after brain hypoxia and ischemia. Curr Opin Clin Nutr Metab Care.

[78] Fiorenzani P, Lamponi S, Magnani A, Ceccarelli I, Aloisi AM. In vitro and in vivo characterization of the new analgesic combination Beta-caryophyllene and docosahexaenoic acid. Evid Based Complement Alternat Med. 2014;596312 doi:10.1155/2014/596312. 10.1155/2014/596312 PMC4109702 25097659

[79] Lin Q, Ruuska SE, Shaw NS, Dong D, Noy N (1999). Ligand selectivity of the peroxisome proliferator-activated receptor α. Biochemistry.

[80] Diep QN, Touyz RM, Schiffrin ELDA (2000). A peroxisome proliferator–activated receptor-α ligand, induces apoptosis in vascular smooth muscle cells by stimulation of p38 mitogen-activated protein kinase. Hypertension.

[81] Strokin M, Sergeeva M, Reiser G (2004). Role of Ca2+−independent phospholipaseA2 and n-3 polyunsaturated fatty acid docosahexaenoic acid in prostanoid production in brain: perspectives for protection in neuroinflammation. Int J Devl Neurosci.

[82] Williams JJ, Mayurasakorn K, Vannucci SJ, Mastropietro C, Bazan NG, Ten VS, Deckelbaum RJ (2013). N-3 fatty acid rich triglyceride emulsions are neuroprotective after cerebral hypoxic-ischemic injury in neonatal mice. PLoS One.

[83] Adibhatla RM, Hatcher JF (2008). Altered lipid metabolism in brain injury and disorders. Subcell Biochem.

[84] Niki E (2009). Lipid peroxidation: physiological levels and dual biological effects. Free Radic Biol Med.

[85] Farkas E, Luiten PG, Bari F (2007). Permanent, bilateral common carotid artery occlusion in the rat: a model for chronic cerebral hypoperfusion-related neurodegenerative diseases. Brain Res Rew.

[86] Molina-Jasso D, Alvarez-González I, Madrigal-Bujaidar E (2009). Clastogenicity of beta-caryophyllene in mouse. Biol Pharm Bull.

